# Supplementary material for: Dynamic updating of clinical survival prediction models in a changing environment
Source: Diagn Progn Res. 2023 Dec 12;7:24. doi: 10.1186/s41512-023-00163-z (PMC10714456; doi:10.1186/s41512-023-00163-z)
Supplement: Supplementary file 1 — Additional file 1. Supplementary information for dynamic updating of clinical survival prediction models in a rapidly changing environment. Details of simulation study data generation, full simulation study results, sensitivity analysis of forgetting factor, QResearch study population characteristics. [file 41512_2023_163_MOESM1_ESM.pdf]

Supplementary Information for Dynamic Updating of Clinical Survival Prediction Models in a Rapidly Changing Environment

by

Kamaryn Tanner[1], Ruth H Keogh[1], Carol AC Coupland[2,3], Julia Hippisley-Cox[2], Karla Diaz-Ordaz[4]

[1]Dept of Medical Statistics, London School of Hygiene and Tropical Medicine, London WC1E 7HT, UK
[2]Nuffield Department of Primary Health Care Sciences, University of Oxford, Oxford OX2 6HT, UK
[3]Centre for Academic Primary Care, School of Medicine, University of Nottingham, Nottingham NG7 2UH, UK
[4]Dept of Statistical Science, University College London, London WC1E 6BT, UK

Appendix A: Details of Bayesian dynamic updating

McCormick et al. [2012] describe a Bayesian dynamic logistic regression technique that we have adapted for proportional hazards regression. Briefly, they posit a relationship between the regression coefficients in period u and those in period $u-1$:

$$\beta_u = \beta_{u-1} + \delta_u \tag{1}$$

where β_u is the vector of regression coefficients in period u and δ_u is a random vector such that $\delta_u \sim N(0, W_u)$. We then assume:

$$\beta_{u-1} \mid (T_{u-1}, X_{u-1}) \sim N(\hat{\beta}_{u-1}, \hat{\Sigma}_{u-1}) \tag{2}$$

where T_{u-1} is the vector of survival times for each individual in the cohort in period $u-1$, X_{u-1} is the matrix of covariates and Σ_{u-1} is a covariance matrix. From the above 2 equations, McCormick et al. [2012] develop the 'prediction equation':

$$\beta_u \mid (T_{u-1}, X_{u-1}) \sim N(\hat{\beta}_{u-1}, \hat{\Sigma}_{u-1} + W_u) \tag{3}$$

By assuming that $W_u \propto \hat{\Sigma}_{u-1}$, Equation 3 becomes:

$$\beta_u \mid (T_{u-1}, X_{u-1}) \sim N(\hat{\beta}_{u-1}, \hat{\Sigma}_{u-1}/\xi_u) \tag{4}$$

where ξ_u is the forgetting factor in period u. Writing this as a Bayesian updating model with exponential survival times, we have:

$$\begin{aligned} T_u &\sim Exp(\omega_u) \\ \omega_u &= \lambda_u + \beta_u^T X_u \\ \beta_u &\sim N(\hat{\beta}_{u-1}, \hat{\Sigma}_{u-1}/\xi_u) \\ \lambda_u &\sim N(0, 2.5) \end{aligned} \tag{5}$$

To start, the original model ($u = 0$) is fit using the initial development dataset with vague priors: $\beta_u \sim N(0, 2.5)$. For models thereafter, estimates from the period $u-1$ model are used to form priors for the period u model. This model is estimated using Markov chain Monte Carlo.

Appendix B: Simulation study data generation

To simulate data used to evaluate and update the prediction model, i.e. data arriving after development of the original model, we used two different data generating mechanisms: *new cohorts* and *cohort with replacement*. These methods are depicted graphically in Figure S1. Details are provided below.

New cohorts data

New cohorts data was generated assuming 1,000 new individuals would join the cohort each month and that the overall follow-up period would be 3 months.

For months $w = 1, \ldots, 15$, where $w = 1$ represents the first month after collection of the original development dataset, data was created as follows:

1. Generate a covariate matrix for n=1,000 unique individuals with $X_1 \sim U(1.8, 9.5)$, $X_2 \sim N(1, 1)$, $X_3 \sim Bern(p_{X_3})$ and $X_4 \sim Bern(p_{X_{4,w}})$ where $p_{X_{4,w}}$ may vary by month. Additionally, p_{X_3} and $p_{X_{4,w}}$ may depend on age (X_1) and on each other as noted in Table S1.

2. For the "New treatment + comorbidity" scenario, we additionally generate an interaction between the new treatment and the comorbidity. Compute $X_5 = X_3 \times X_4$

3. Given log hazard ratios $\beta_1, \beta_2, \beta_3, \beta_4, \beta_5$, and assuming exponentially distributed survival times with baseline rate parameter λ_w, the hazard of an event at time t can be written $h(t) = \lambda_w \exp(\beta_1 X_1 + \beta_2 X_2 + \beta_3 X_3 + \beta_4 X_4 + \beta_5 X_5)$. Survival times T are generated using the cumulative hazard inversion technique [Bender et al., 2005] and then administrative censoring is applied for each person at t=0.25 (3 months), t=0.167 (2 months) or t=0.083 (1 month) if they joined the cohort in the first, second or third month of the quarter, respectively. If $T <$ censoring time, set the event indicator E to 1, otherwise 0.

These monthly datasets were combined to form quarterly datasets, each with n =3,000 records. Within each quarterly dataset, one-third of the individuals were followed up for 3-months, one-third for 2-months and one-third for one-month. See Figure S1-top.

Cohort with replacement data

Generation of cohort with replacement data required a different procedure. First, a month 1 covariate matrix was generated for 11,000 individuals. As our aim was to have 10,000 people at the start of each period, data was generated for 10,000 people in the cohort at the beginning plus an additional 1,000 extras used to replace those individuals who had events. The covariate matrix is comprised of: $X_1 \sim U(1.8, 9.5)$, $X_2 \sim N(1, 1)$, $X_3 \sim Bern(p_{X_3})$ and $X_4 \sim Bern(p_{X_{4,1}})$. X_1, X_2, X_3 remain fixed for all months. For the "New treatment + comorbidity" scenario, we also generate the interaction between the comorbidity and the treatment, $X_{5,1} = X_3 \times X_{4,1}$. For months $w = 2, \ldots, 15$:

1. Set $X_{4,w} = 1$ for all individuals with $X_{4,(w-1)} = 1$ to indicate that once an individual is treated, they remain treated in all future months.

2. Based on $p_{X_{4,w}}$ and other constraints (see Supplementary Information Table S1), set $X_{4,w} = 1$ for a random selection of eligible individuals who were previously untreated, ie. $X_{4,w-1} = 0$. This is used to simulate the introduction of a new treatment that is rolled out to an increasing number of people over time.

3. For the “New treatment + comorbidity” scenario, compute $X_{5,w} = X_3 \times X_{4,w}$

4. Given log hazard ratios $\beta_1, \beta_2, \beta_3, \beta_4, \beta_5$, and assuming exponentially distributed survival times with baseline rate parameter λ_w, the hazard of an event at time t can be written $h(t) = \lambda_w \exp(\beta_1 X_1 + \beta_2 X_2 + \beta_3 X_3 + \beta_4 X_4 + \beta_5 X_5)$. Survival times T are generated using the cumulative hazard inversion technique [Bender et al., 2005] and then administrative censoring is applied for each person at t=0.25 (3 months), t=0.167 (2 months) or t=0.083 (1 month) if they joined the cohort in the first, second or third month of the quarter, respectively. If T <0.25, set the event indicator E to 1, otherwise 0.

Number of simulated datasets

Our aim was to detect a difference of 0.01 in the mean c-index with a 5% significance level and 90% power. Using a standard formula based on the normal distribution and assuming a C-index standard deviation of less than 0.05, we need at least 525 simulated datasets. To be conservative, we used n_{sim}=600 repetitions.

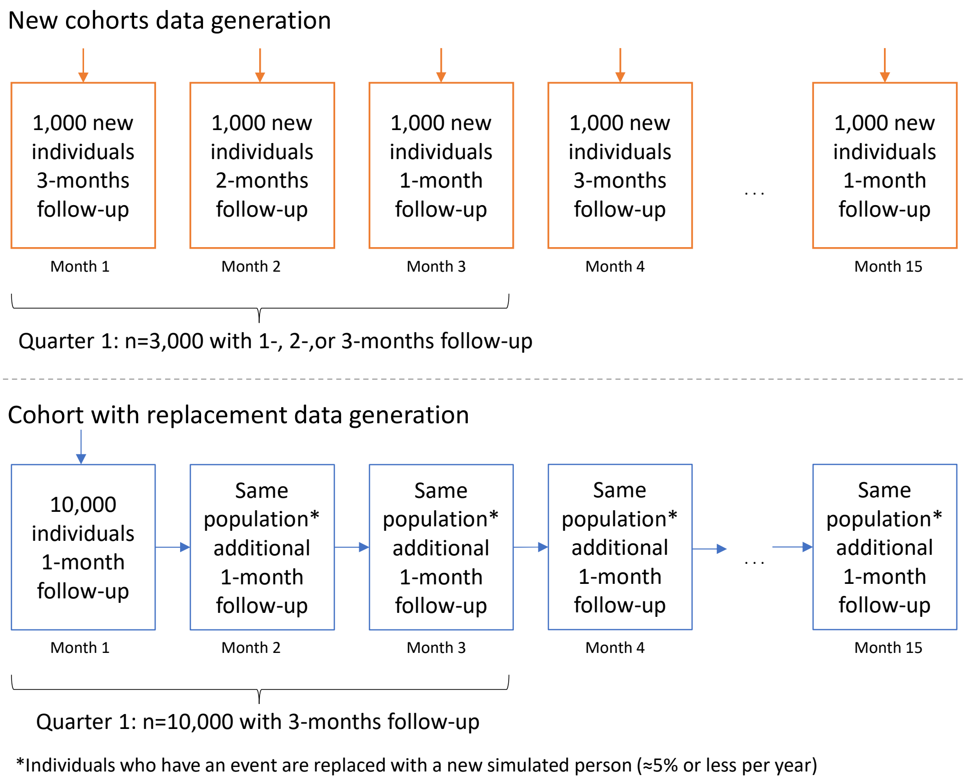

Figure S1: Illustration of the two simulated data generation processes. To generate *new cohorts* simulation data (top), new individuals are simulated each month with 3-, 2- or 1-month of follow-up. This method mimics the situation where individuals have had a certain test result, diagnosis or experience that makes them eligible to be in the modelled population. Generation of *cohort with replacement* data (bottom) begins by simulating an initial population of 10,000 individuals who have additional follow-up time added each month. When individuals experience an event, a new simulated individual is added to replace them. This is similar to data that would be retrieved from electronic health records where the population is essentially fixed but there is some small amount of churn.

Table S1: Simulation parameters by scenario. λ is the baseline rate parameter, βs are log hazard ratios and p_{X_a} is probability(X_a=1).

Scenario	Baseline rate (λ)	log HR (β)	Value of p
All scenarios		β_1 =0.50	-
		β_2 =0.75	-
Reference scenario	λ_w =0.0007	β_3 =-0.25	p_{X_3} =0.35
	$w = 1, \ldots, 15$	β_4, β_5 =NA	p_{X_4} =NA
Scenarios with calibration drift			
Decreasing events	decreasing, range=	β_3 =-0.25	p_{X_3} =0.35
	λ_{month1} =0.0007	β_4 =NA	p_{X_4} =NA
	λ_{month15} =0.00025	β_5 =NA	
Increasing events	increasing, range=	β_3 =-0.25	p_{X_3} =0.35
	λ_{month1} =0.00072	β_4 =NA	p_{X_4} =NA
	λ_{month15} =0.00137	β_5 =NA	
Scenario with rare predictor			
Rare predictor	λ_w =0.00065	β_3 =0.8	p_{X_3} increases with X_1
	$w = 1, \ldots, 15$		p_{X_3} =0.005 if 5.5$\leq X_1$ <6.0
			p_{X_3} =0.005 if 6.0$\leq X_1$ <6.5
			p_{X_3} =0.01 if 6.5$\leq X_1$ <7.0
			p_{X_3} =0.02 if 7.0$\leq X_1$ <7.5
			p_{X_3} =0.02 if 7.5$\leq X_1$ <8.0
			p_{X_3} =0.03 if 8.0$\leq X_1$ <8.5
			p_{X_3} =0.04 if 8.5$\leq X_1$ <9.0
			p_{X_3} =0.05 if 9.0$\leq X_1$
		β_4 =NA	p_{X_4} =NA
Scenarios with new predictors			
New treatment	λ_w =0.001	β_3 =NA	p_{X_3} =NA
	$w = 1, \ldots, 15$	β_4 =-2.3	p_{X_4} increases with time, X_1
			$p_{X_{4,4}}$ =0.05 (8.0$\leq X_1$)
			$p_{X_{4,5}}$ =0.10 (8.0$\leq X_1$)
			$p_{X_{4,6}}$ =0.15 (7.5$\leq X_1$)
			$p_{X_{4,7}}$ =0.18 (7.0$\leq X_1$)
			$p_{X_{4,8}}$ =0.24 (7.0$\leq X_1$)
			$p_{X_{4,9}}$ =0.30 (6.5$\leq X_1$)
			$p_{X_{4,10}}$ =0.33 (6.0$\leq X_1$)
			$p_{X_{4,11}}$ =0.37 (6.0$\leq X_1$)
			$p_{X_{4,12}}$ =0.45 (5.0$\leq X_1$)
			$p_{X_{4,13}}$ =0.50 (5.0$\leq X_1$)
			$p_{X_{4,14}}$ =0.60 (3.0$\leq X_1$)
			$p_{X_{4,15}}$ =0.675 (3.0$\leq X_1$)
New treatment +	decreasing, range=	β_3 =1.78	p_{X_3} =0.05
comorbidity	λ_1 =0.00060	β_4 =-1.77	$p_{X_{4,4}}$ =0.02 (8.0$\leq X_1$ or $X_3 = 1$)
	λ_{15} =0.00046	β_5 =0.26	$p_{X_{4,5}}$ =0.04 (8.0$\leq X_1$ or $X_3 = 1$)
			$p_{X_{4,6}}$ =0.07 (8.0$\leq X_1$ or $X_3 = 1$)
			$p_{X_{4,7}}$ =0.11 (7.0$\leq X_1$ or $X_3 = 1$)
			$p_{X_{4,8}}$ =0.16 (7.0$\leq X_1$ or $X_3 = 1$)
			$p_{X_{4,9}}$ =0.25 (7.0$\leq X_1$ or $X_3 = 1$)
			$p_{X_{4,10}}$ =0.325 (6.0$\leq X_1$ or $X_3 = 1$)
			$p_{X_{4,11}}$ =0.40 (6.0$\leq X_1$ or $X_3 = 1$)
			$p_{X_{4,12}}$ =0.475 (5.0$\leq X_1$ or $X_3 = 1$)
			$p_{X_{4,13}}$ =0.525 (5.0$\leq X_1$ or $X_3 = 1$)
			$p_{X_{4,14}}$ =0.55 (3.0$\leq X_1$ or $X_3 = 1$)
			$p_{X_{4,15}}$ =0.575 (3.0$\leq X_1$ or $X_3 = 1$)

Appendix C: Simulation study results

Table S2: Simulation results for the reference scenario with constant baseline hazard for both cohort with replacement (left) and new cohorts (right) simulated datasets. For each updating method and dataset type, the C-index, calibration intercept, calibration slope, and Brier score over 600 simulated datasets is shown for a model updated quarterly over one year. Monte Carlo standard error was <0.005 for all data points. Bold text indicates that a method was significantly better than all other methods ($p < 0.05$) using a Wilcoxon signed rank test.

**Cohort with replacement/Reference scenario**					**New Cohorts/Reference scenario**				
	Predictions on data from:					Predictions on data from:			
C-index	Q2	Q3	Q4	Q5	C-index	Q2	Q3	Q4	Q5
No update	0.81	0.81	0.81	0.81	No update	0.81	0.82	0.81	0.81
Recal always	0.81	0.81	0.81	0.81	Recal always	0.81	0.82	0.81	0.81
Refit always	0.81	0.81	0.81	0.81	Refit always	0.80	0.81	0.80	0.81
Bayesian	0.81	0.81	0.81	0.81	Bayesian	0.81	0.82	0.81	0.81
Calibration intercept	Q2	Q3	Q4	Q5	Calibration intercept	Q2	Q3	Q4	Q5
No update	-0.01	0.00	-0.01	-0.01	No update	-0.01	0.01	-0.02	-0.02
Recal always	-0.02	-0.01	-0.03	-0.02	Recal always	0.02	0.04	-0.02	0.04
Refit always	-0.01	0.01	-0.01	0.00	Refit always	0.00	0.02	-0.04	0.01
Bayesian	0.00	0.02	-0.01	0.00	Bayesian	0.02	0.04	-0.02	0.03
Calibration slope	Q2	Q3	Q4	Q5	Calibration slope	Q2	Q3	Q4	Q5
No update	1.00	0.99	1.01	1.00	No update	1.00	1.02	1.01	1.01
Recal always	1.00	0.99	1.01	1.00	Recal always	1.00	1.02	1.01	1.01
Refit always	1.00	0.99	1.00	0.99	Refit always	0.96	0.98	0.96	0.97
Bayesian	1.00	**1.00**	1.01	**1.00**	Bayesian	1.00	1.02	**1.01**	1.01
Brier score	Q2	Q3	Q4	Q5	Brier score	Q2	Q3	Q4	Q5
No update	0.01	0.01	0.01	0.01	No update	0.01	0.01	0.01	0.01
Recal always	0.01	0.01	0.01	0.01	Recal always	0.01	0.01	0.01	0.01
Refit always	0.01	0.01	0.01	0.01	Refit always	0.01	0.01	0.01	0.01
Bayesian	0.01	0.01	0.01	0.01	Bayesian	0.01	0.01	0.01	0.01

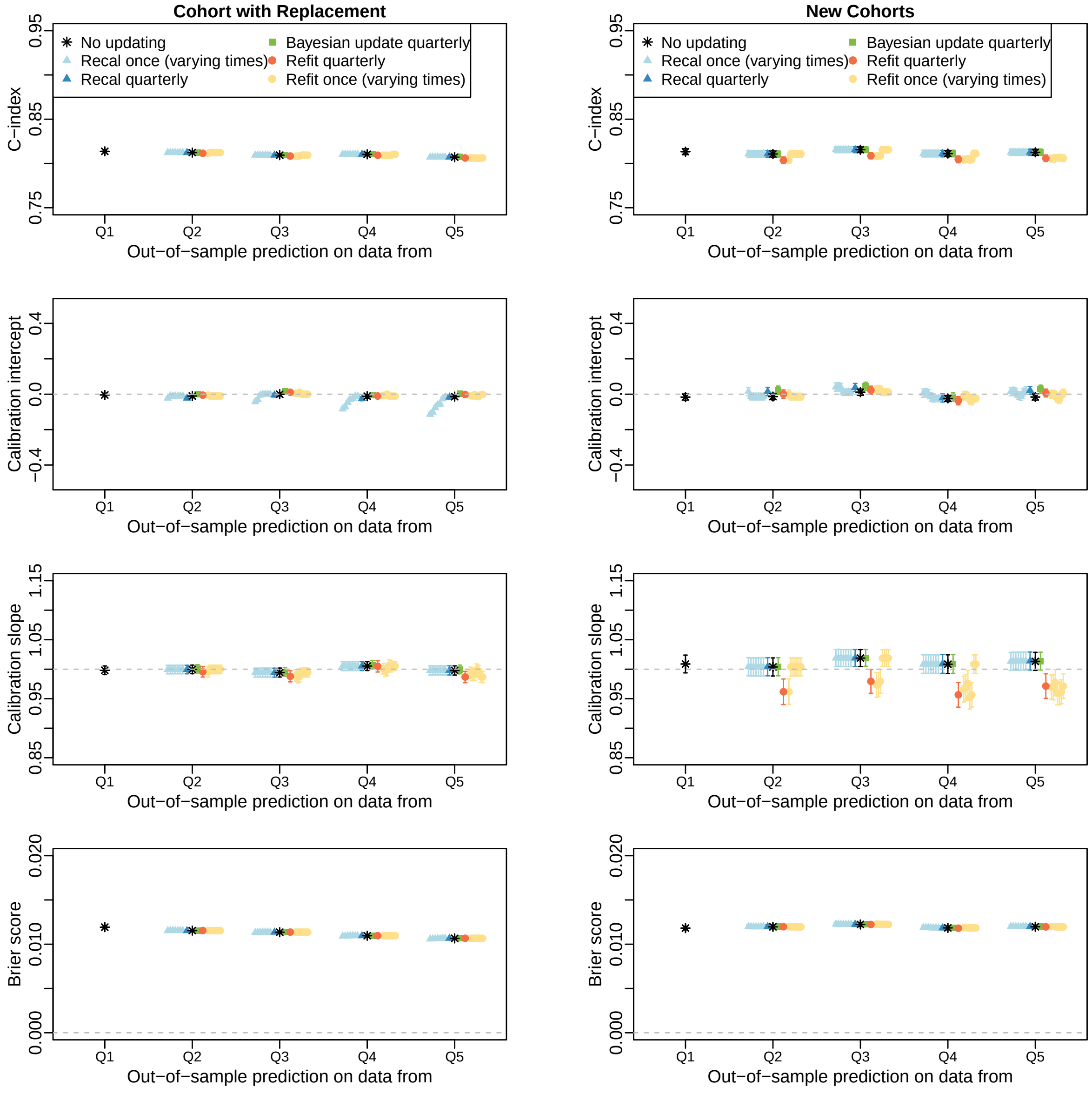

Figure S2: Simulation results for the reference scenario with constant baseline hazard for both cohort with replacement (left) and new cohorts (right) simulated datasets. Best possible values for calibration intercept, calibration slope and Brier score are indicated with gray dashed lines. Results for 'Recal once' and 'Refit once' strategies are ordered by update time with the earliest time on the left.

Table S3: Simulation results for the calibration drift scenario with decreasing event rate for both cohort with replacement (left) and new cohorts (right) simulated datasets. For each updating method and dataset type, the C-index, calibration intercept, calibration slope, and Brier score over 600 simulated datasets is shown for a model updated quarterly over one year. Monte Carlo standard error was <0.005 for all data points. Bold text indicates that a method was significantly better than all other methods ($p < 0.05$) for that evaluation metric using a Wilcoxon signed rank test.

**Cohort with replacement/Decreasing event rate**					**New Cohorts/Decreasing event rate**				
	Predictions on data from:					Predictions on data from:			
C-index	Q2	Q3	Q4	Q5	C-index	Q2	Q3	Q4	Q5
No update	0.81	0.81	0.81	0.81	No update	0.81	0.82	0.81	0.82
Recal always	0.81	0.81	0.81	0.81	Recal always	0.81	0.82	0.81	0.82
Refit always	0.81	0.81	0.81	0.81	Refit always	0.80	0.81	0.80	0.81
Bayesian	**0.81**	**0.81**	0.81	**0.81**	Bayesian	0.81	0.82	0.81	0.82
Calibration intercept	Q2	Q3	Q4	Q5	Calibration intercept	Q2	Q3	Q4	Q5
No update	-0.03	-0.22	-0.49	-0.88	No update	-0.02	-0.19	-0.49	-0.88
Recal always	-0.04	-0.20	-0.29	-0.40	Recal always	0.00	-0.15	-0.29	-0.38
Refit always	-0.03	-0.18	-0.28	-0.39	Refit always	-0.01	-0.17	-0.30	-0.40
Bayesian	-0.02	**-0.18**	**-0.27**	**-0.38**	Bayesian	0.01	-0.15	**-0.28**	**-0.36**
Calibration slope	Q2	Q3	Q4	Q5	Calibration slope	Q2	Q3	Q4	Q5
No update	1.00	0.99	1.01	1.00	No update	1.00	1.02	1.01	1.05
Recal always	1.00	0.99	1.01	1.00	Recal always	1.00	1.02	1.01	1.05
Refit always	1.00	0.98	1.01	0.99	Refit always	0.96	0.98	0.95	0.99
Bayesian	1.00	**0.99**	**1.01**	**1.00**	Bayesian	1.00	**1.02**	1.01	1.05
Brier score	Q2	Q3	Q4	Q5	Brier score	Q2	Q3	Q4	Q5
No update	0.01	0.01	0.01	0.00	No update	0.01	0.01	0.01	0.01
Recal always	0.01	0.01	0.01	0.00	Recal always	0.01	0.01	0.01	0.01
Refit always	0.01	0.01	0.01	0.00	Refit always	0.01	0.01	0.01	0.01
Bayesian	0.01	0.01	0.01	0.00	Bayesian	0.01	0.01	0.01	0.01

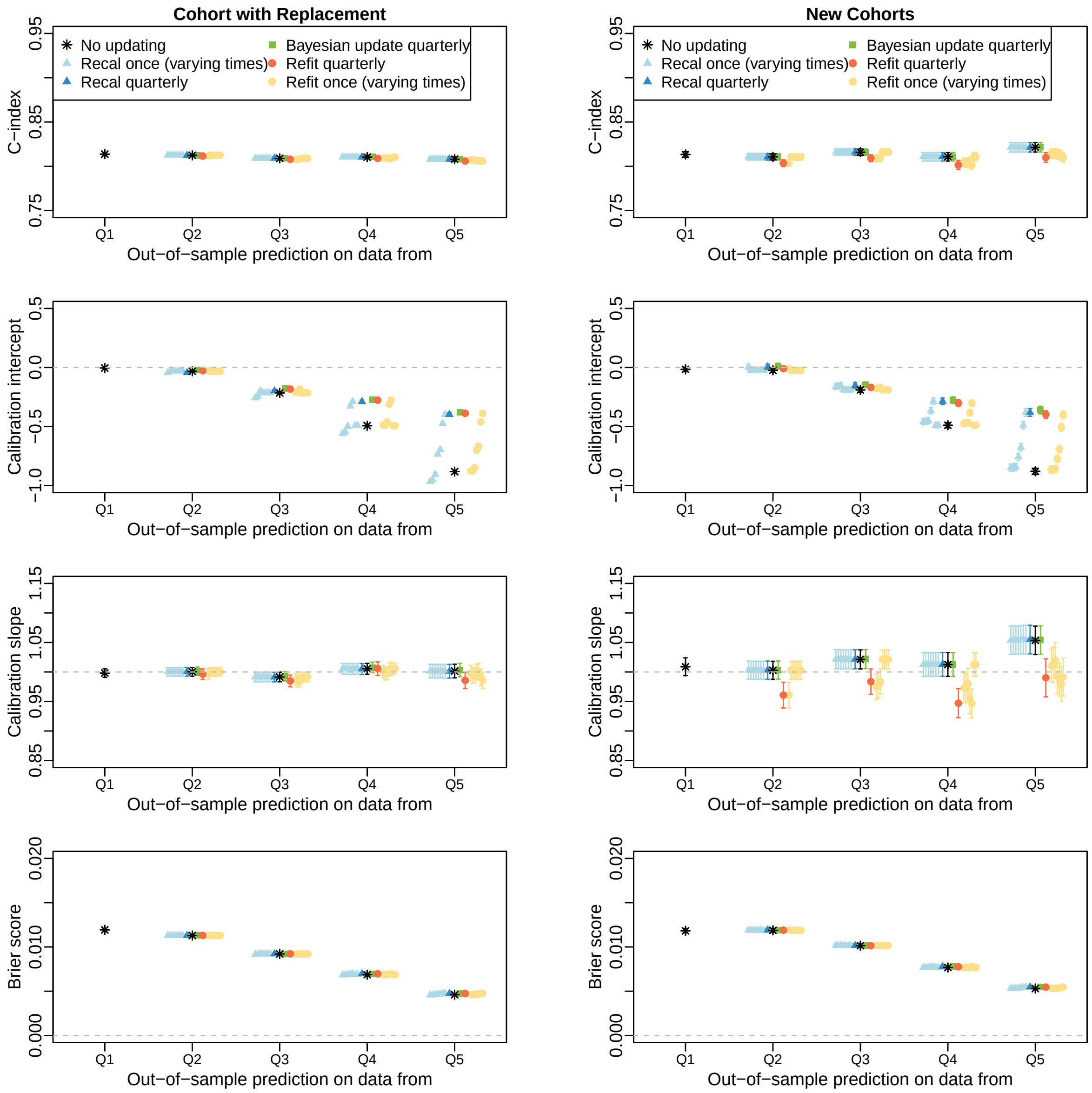

Figure S3: Simulation results for the calibration drift scenario with decreasing event rate for both cohort with replacement (left) and new cohorts (right) simulated datasets. Best possible values for calibration intercept, calibration slope and Brier score are indicated with gray dashed lines. Results for 'Recal once' and 'Refit once' strategies are ordered by update time with the earliest time on the left.

Table S4: Simulation results for the calibration drift scenario with increasing event rate for both open cohort (left) and new cohorts (right) simulated datasets. For each updating method and dataset type, the C-index, calibration intercept, calibration slope, and Brier score over 600 simulated datasets is shown for a model updated quarterly over one year. Monte Carlo standard error was <0.005 for all data points. Bold text indicates that a method was significantly better than all other methods ($p < 0.05$) using a Wilcoxon signed rank test.

**Cohort with replacement/Increasing event rate**					**New cohorts/Increasing event rate**				
	Predictions on data from:					Predictions on data from:			
C-index	Q2	Q3	Q4	Q5	C-index	Q2	Q3	Q4	Q5
No update	0.81	0.81	0.81	0.81	No update	0.81	0.81	0.81	0.82
Recal always	0.81	0.81	0.81	0.81	Recal always	0.81	0.81	0.81	0.82
Refit always	0.81	0.81	0.81	0.81	Refit always	0.80	0.81	0.81	0.81
Bayesian	**0.81**	**0.81**	**0.81**	**0.81**	Bayesian	0.81	**0.81**	**0.81**	0.82
Calibration intercept	Q2	Q3	Q4	Q5	Calibration intercept	Q2	Q3	Q4	Q5
No update	0.09	0.36	0.49	0.60	No update	0.03	0.23	0.32	0.41
Recal always	0.08	**0.25**	**0.11**	**0.09**	Recal always	0.06	0.21	0.11	0.10
Refit always	0.10	0.27	0.13	0.11	Refit always	0.04	0.20	0.09	0.08
Bayesian	0.10	0.27	0.13	0.11	Bayesian	0.06	0.22	0.11	0.10
Calibration slope	Q2	Q3	Q4	Q5	Calibration slope	Q2	Q3	Q4	Q5
No update	1.00	1.00	1.00	1.00	No update	1.00	1.01	1.01	1.01
Recal always	1.00	1.00	1.01	1.00	Recal always	1.00	1.01	1.01	1.01
Refit always	0.99	0.99	1.00	0.99	Refit always	0.96	0.98	0.97	0.98
Bayesian	1.00	1.00	1.01	1.00	Bayesian	1.00	1.01	1.01	1.01
Brier score	Q2	Q3	Q4	Q5	Brier score	Q2	Q3	Q4	Q5
No update	0.01	0.02	0.02	0.02	No update	0.01	0.02	0.02	0.02
Recal always	**0.01**	**0.02**	**0.02**	**0.02**	Recal always	0.01	0.02	0.02	0.02
Refit always	0.01	0.02	0.02	0.02	Refit always	0.01	0.02	0.02	0.02
Bayesian	0.01	0.02	0.02	0.02	Bayesian	0.01	0.02	0.02	0.02

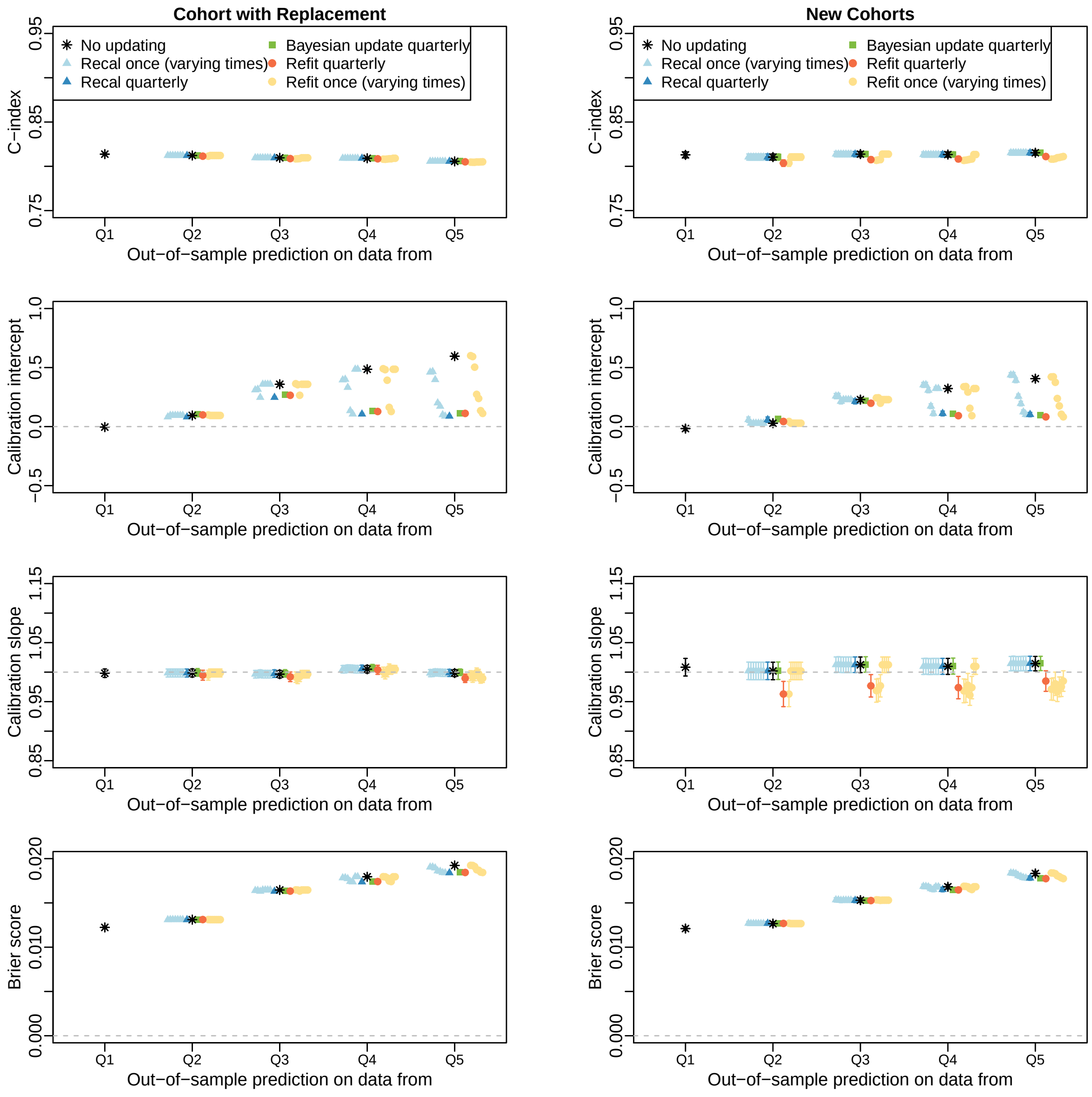

Figure S4: Simulation results for the calibration drift scenario with increasing event rate for both cohort with replacement (left) and new cohorts (right) simulated datasets. Best possible values for calibration intercept, calibration slope and Brier score are indicated with gray dashed lines. Results for 'Recal once' and 'Refit once' strategies are ordered by update time with the earliest time on the left.

Table S5: Simulation results for rare predictor scenario with 1% having a risk factor for an event for both cohort with replacement (left) and new cohorts (right) simulated datasets. For each updating method and dataset type, the C-index, calibration intercept and slope, and Brier score over 600 simulated datasets is shown for a model updated quarterly over one year. Monte Carlo standard error was <0.005 for all data points. Bold text indicates that a method was significantly better than all other methods ($p < 0.05$) for that evaluation metric using a Wilcoxon signed rank test.

Open Cohort/Rare-1%

	Predictions on data from:			
C-index	Q2	Q3	Q4	Q5
No update	0.82	0.81	0.81	0.81
Recal always	0.82	0.81	0.81	0.81
Refit always	0.81	0.81	0.81	0.81
Bayesian	**0.82**	**0.81**	**0.81**	**0.81**
Calibration intercept	Q2	Q3	Q4	Q5
No update	0.00	-0.00	-0.01	-0.00
Recal always	-0.01	-0.02	-0.02	-0.01
Refit always	0.01	-0.01	-0.00	0.01
Bayesian	0.02	-0.00	-0.00	0.01
Calibration slope	Q2	Q3	Q4	Q5
No update	1.00	1.00	1.00	1.00
Recal always	1.00	1.00	1.00	1.00
Refit always	1.00	1.00	0.99	0.99
Bayesian	1.00	1.01	1.00	1.00
Brier score	Q2	Q3	Q4	Q5
No update	0.01	0.01	0.01	0.01
Recal always	0.01	0.01	0.01	0.01
Refit always	0.01	0.01	0.01	0.01
Bayesian	0.01	0.01	0.01	0.01

New Cohorts/Rare-1%

	Predictions on data from:			
C-index	Q2	Q3	Q4	Q5
No update	0.82	0.82	0.81	0.82
Recal always	0.82	0.82	0.82	0.81
Refit always	0.81	0.81	0.81	0.81
Bayesian	0.82	0.82	0.82	0.81
Calibration intercept	Q2	Q3	Q4	Q5
No update	0.00	0.00	0.00	0.00
Recal always	0.02	0.01	0.02	0.01
Refit always	-0.02	-0.02	-0.02	-0.01
Bayesian	0.03	0.02	0.02	0.02
Calibration slope	Q2	Q3	Q4	Q5
No update	1.00	1.00	0.99	0.99
Recal always	1.01	1.01	0.99	0.99
Refit always	0.97	0.97	0.94	0.95
Bayesian	1.01	1.01	0.99	0.99
Brier score	Q2	Q3	Q4	Q5
No update	0.01	0.01	0.01	0.01
Recal always	0.01	0.01	0.01	0.01
Refit always	0.01	0.01	0.01	0.01
Bayesian	0.01	0.01	0.01	0.01

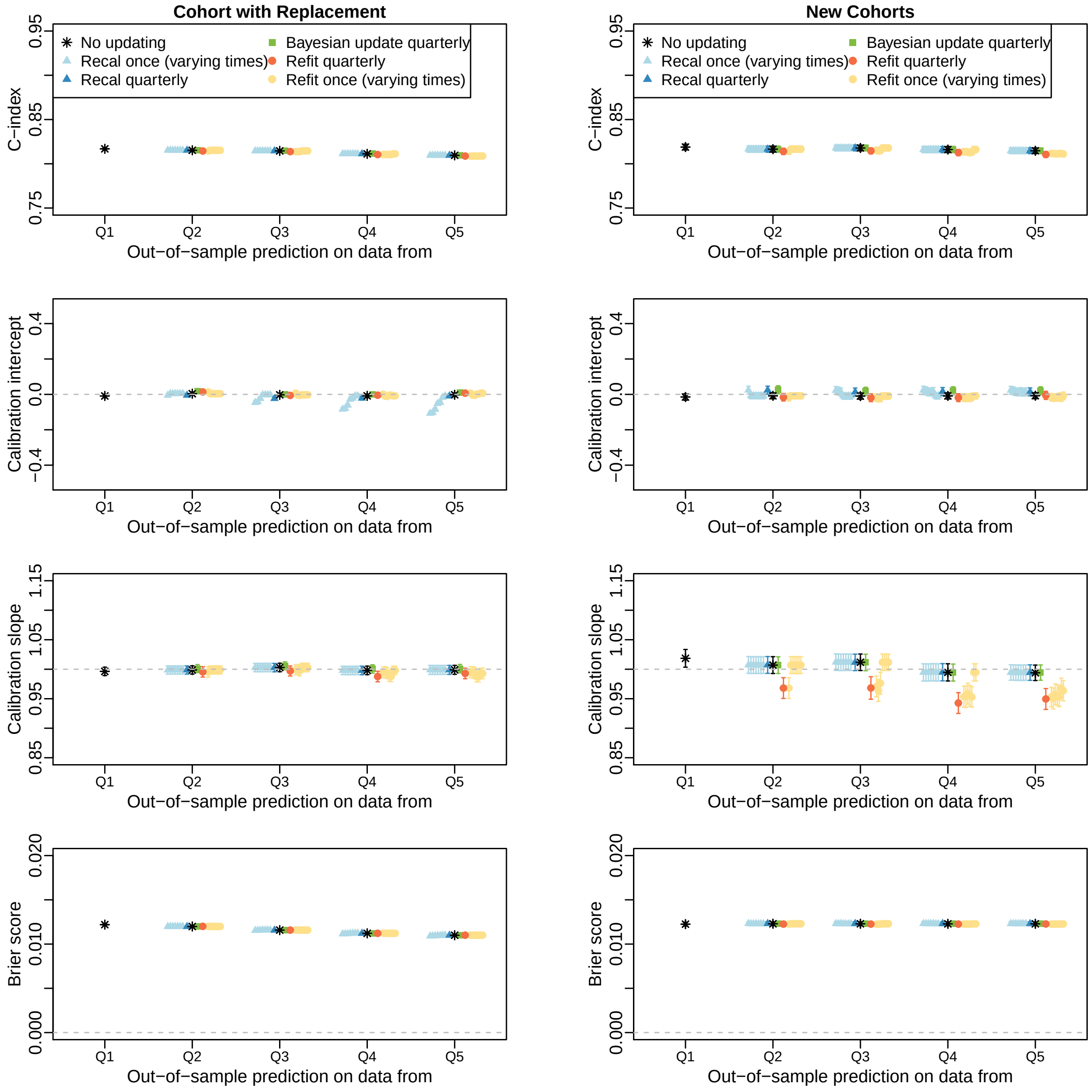

Figure S5: Simulation results for the rare predictor scenario where 1% of the population is at increased risk for an event. Cohort with replacement (left) and new cohorts (right) simulated datasets are shown. Best possible values for calibration intercept, calibration slope and Brier score are indicated with gray dashed lines. Results for 'Recal once' and 'Refit once' strategies are ordered by update time with the earliest time on the left.

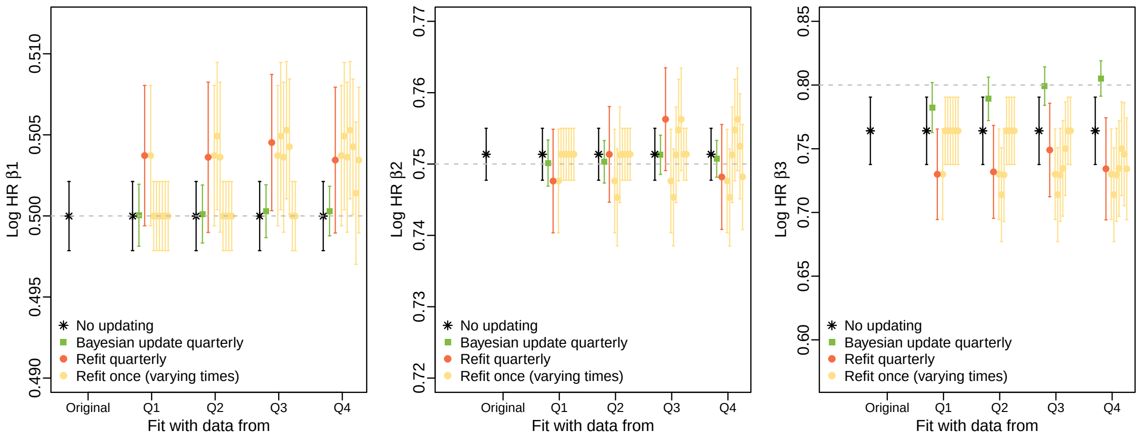

Figure S6: Average estimated log hazard ratio (HR) estimates in the Rare-1% scenario with a cohort with replacement. The true log hazard ratio is shown by the gray dashed line. Results for 'Refit once' strategies are ordered by update time with the earliest time on the left. Intercept recalibration strategies are not shown because hazard ratios are not re-estimated.

Table S6: Simulation results for the new treatment scenario for both cohort with replacement (left) and new cohorts (right) simulated datasets. For each updating method and dataset type, the C-index, calibration intercept, calibration slope, and Brier score over 600 simulated datasets is shown for a model updated quarterly over one year. Monte Carlo standard error was <0.005 for all data points. Bold text indicates that a method was significantly better than all other methods ($p < 0.05$) for that evaluation metric using a Wilcoxon signed rank test.

**Cohort with replacement/New treatment**					**New Cohorts/New treatment**				
	Predictions on data from:					Predictions on data from:			
C-index	Q2	Q3	Q4	Q5	C-index	Q2	Q3	Q4	Q5
No update	0.78	0.73	0.68	0.66	No update	0.79	0.76	0.74	0.75
Recal always	0.78	0.73	0.68	0.66	Recal always	0.79	0.76	0.74	0.75
Refit always	0.78	0.75	0.73	0.73	Refit always	0.79	0.80	0.81	0.82
Bayesian	**0.78**	**0.75**	0.72	0.72	Bayesian	0.79	**0.81**	**0.82**	**0.83**
Calibration intercept	Q2	Q3	Q4	Q5	Calibration intercept	Q2	Q3	Q4	Q5
No update	-0.26	-0.67	-1.08	-1.56	No update	-0.24	-0.58	-0.92	-1.18
Recal always	-0.30	-0.42	-0.41	-0.48	Recal always	-0.21	-0.32	-0.33	-0.25
Refit always	-0.27	-0.17	**-0.17**	**-0.20**	Refit always	-0.24	0.01	-0.02	-0.01
Bayesian	-0.27	**-0.16**	-0.19	-0.23	Bayesian	-0.22	0.02	0.00	0.01
Calibration slope	Q2	Q3	Q4	Q5	Calibration slope	Q2	Q3	Q4	Q5
No update	0.85	0.66	0.50	0.48	No update	0.89	0.78	0.74	0.81
Recal always	0.85	0.66	0.50	0.48	Recal always	0.89	0.78	0.74	0.81
Refit always	0.85	0.93	**0.91**	**0.95**	Refit always	0.89	0.75	0.87	0.91
Bayesian	0.85	**0.93**	0.85	0.83	Bayesian	0.89	**1.00**	**1.00**	**0.99**
Brier score	Q2	Q3	Q4	Q5	Brier score	Q2	Q3	Q4	Q5
No update	0.01	0.01	0.01	0.00	No update	0.01	0.01	0.01	0.01
Recal always	0.01	0.01	0.01	0.00	Recal always	0.01	0.01	0.01	0.01
Refit always	0.01	0.01	0.01	0.00	Refit always	0.01	0.01	0.01	0.01
Bayesian	0.01	0.01	0.01	0.00	Bayesian	0.01	0.01	0.01	0.01

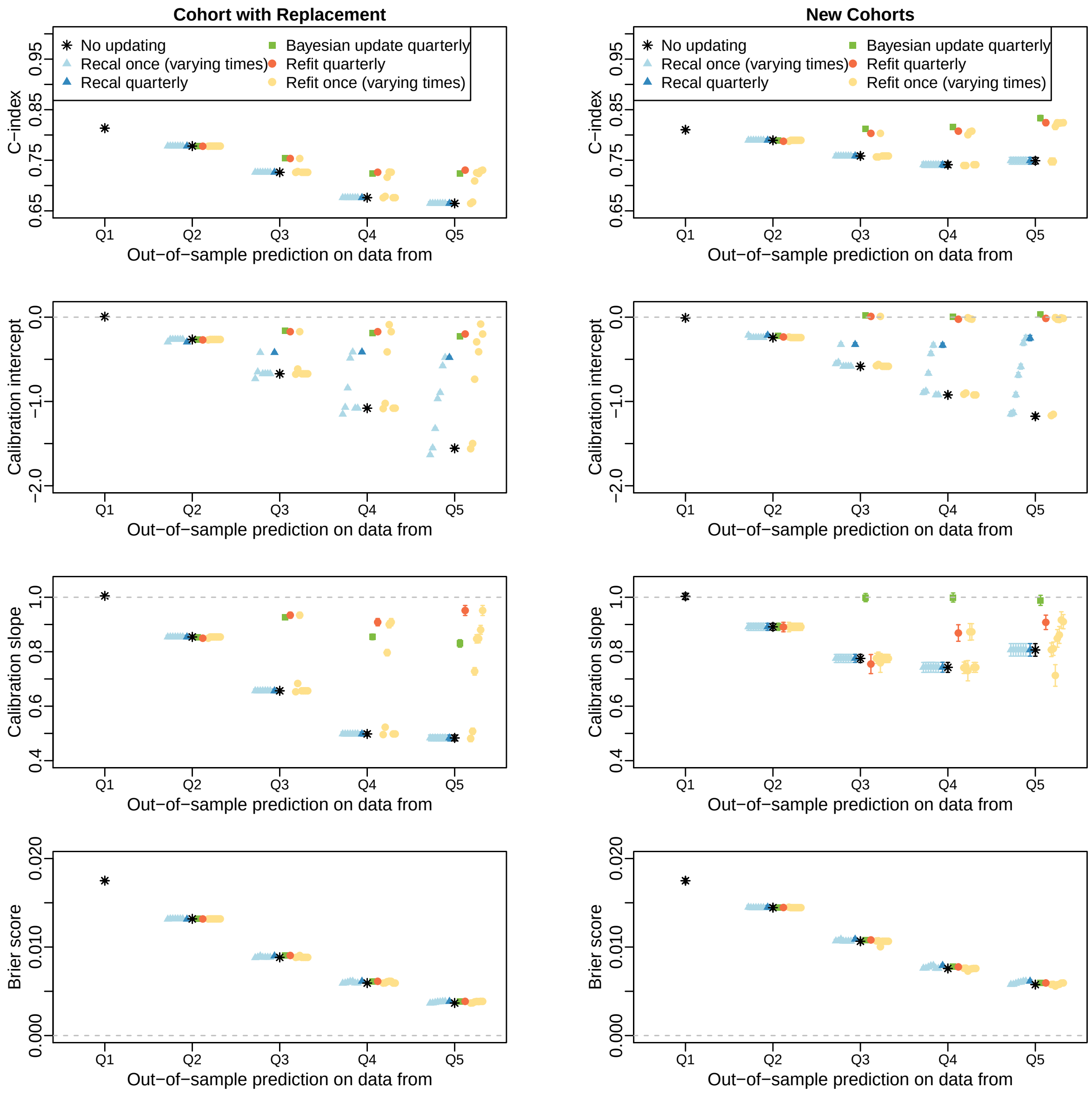

Figure S7: Simulation results for the scenario where a new treatment was introduced in Q2. On the left, results for the cohort with replacement simulation; on the right, results for the new cohorts simulation. Best possible values for calibration intercept, calibration slope and Brier score are indicated with gray dashed lines. Results for 'Recal once' and 'Refit once' strategies are ordered by update time with the earliest time on the left.

Table S7: Simulation results for the new treatment + comorbidity scenario for both cohort with replacement (left) and new cohorts (right) simulated datasets. For each updating method and dataset type, the C-index, calibration intercept, calibration slope, and Brier score over 600 simulated datasets is shown for a model updated quarterly over one year. Monte Carlo standard error was <0.005 for all data points. Bold text indicates that a method was significantly better than all other methods ($p < 0.05$) for that evaluation metric using a Wilcoxon signed rank test.

**Cohort with replacement/New trt + comorbidity**					**New Cohorts/New trt + comorbidity**				
	Predictions on data from:					Predictions on data from:			
C-index	Q2	Q3	Q4	Q5	C-index	Q2	Q3	Q4	Q5
No update	0.82	0.79	0.74	0.72	No update	0.83	0.81	0.78	0.80
Recal always	0.82	0.79	0.74	0.72	Recal always	0.83	0.81	0.78	0.80
Refit always	0.82	0.79	0.76	0.76	Refit always	0.82	0.81	0.78	0.81
Bayesian	**0.82**	**0.79**	**0.76**	**0.76**	Bayesian	0.83	**0.83**	**0.81**	**0.83**
Calibration intercept	Q2	Q3	Q4	Q5	Calibration intercept	Q2	Q3	Q4	Q5
No update	-0.16	-0.51	-1.11	-1.50	No update	-0.16	-0.47	-1.03	-1.22
Recal always	-0.18	-0.36	-0.61	-0.40	Recal always	-0.10	-0.29	-0.55	-0.18
Refit always	-0.15	-0.12	-0.35	-0.42	Refit always	-0.14	-0.45	-0.85	-0.76
Bayesian	**-0.14**	**-0.09**	**-0.33**	**-0.28**	Bayesian	-0.11	**-0.04**	**-0.01**	**0.02**
Calibration slope	Q2	Q3	Q4	Q5	Calibration slope	Q2	Q3	Q4	Q5
No update	0.96	0.85	0.69	0.68	No update	0.98	0.90	0.82	0.91
Recal always	0.96	0.85	0.69	0.67	Recal always	0.98	0.90	0.82	0.91
Refit always	0.96	0.94	0.89	0.93	Refit always	0.94	0.87	0.82	0.89
Bayesian	0.96	**0.95**	**0.89**	**0.93**	Bayesian	0.98	**0.96**	**0.91**	**0.95**
Brier score	Q2	Q3	Q4	Q5	Brier score	Q2	Q3	Q4	Q5
No update	0.01	0.01	0.00	0.00	No update	0.01	0.01	0.01	0.00
Recal always	0.01	0.01	0.00	0.00	Recal always	0.01	0.01	0.01	0.00
Refit always	0.01	0.01	0.00	0.00	Refit always	0.01	0.01	0.01	0.00
Bayesian	0.01	0.01	0.00	0.00	Bayesian	0.01	0.01	0.01	0.00

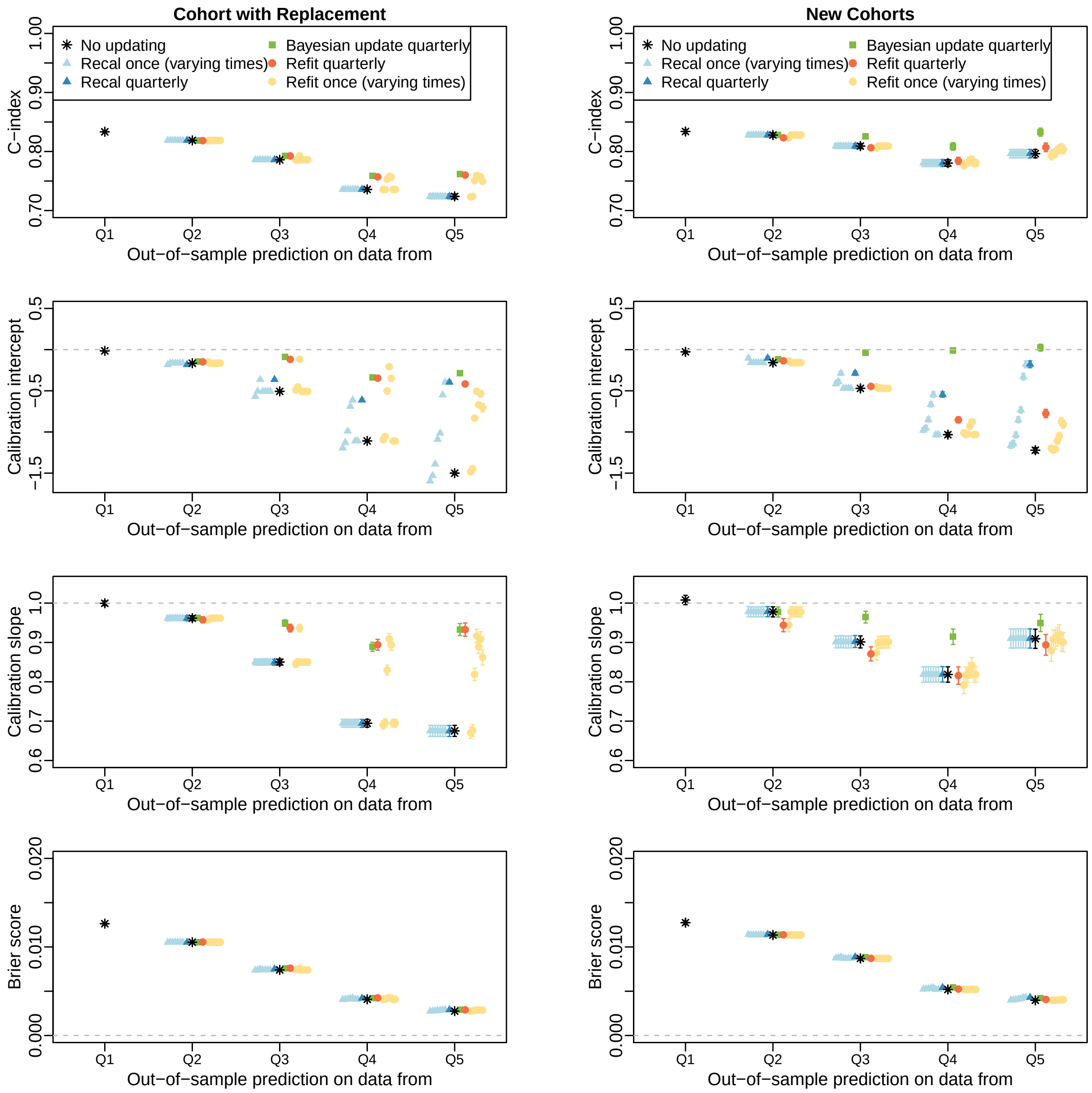

Figure S8: Simulation results for the new treatment + comorbidity scenario. On the left, results for the cohort with replacement simulation; on the right, results for the new cohorts simulation. Best possible values for calibration intercept, calibration slope and Brier score are indicated with gray dashed lines. Results for 'Recal once' and 'Refit once' strategies are ordered by update time with the earliest time on the left.

Appendix D: Sensitivity analysis of the forgetting factor

We selected two scenarios to investigate the impact of different values of ξ, the forgetting factor in Bayesian dynamic updating. Table S8 shows the performance over time of Bayesian updating with ξ= 0.5, 0.75, 0.9 and 0.99 in the new treatment+comorbidity scenario (left) and decreasing events scenario (right). For both scenarios, the analysis was insensitive to the choice of ξ for values of 0.5, 0.75, 0.9, and 0.99 with no significant differences seen for C-index, calibration intercept and slope, or Brier score.

Table S8: Simulation results for the new treatment + comorbidity scenario (left) and decreasing event rate scenario (right) when the Bayesian dynamic updating used different forgetting factors (FF). A forgetting factor close to 1 equates to less uncertainty in the prior used in the updated Bayesian model. For each updating method and dataset type, the C-index, calibration intercept, calibration slope, and Brier score over 600 simulated datasets is shown for a model updated quarterly over one year. Monte Carlo standard error was <0.005 for all data points. New cohorts-style simulation datasets were used.

**New Cohorts/New treatment+comorbidity**					**New Cohorts/Decreasing event rate**				
	Predictions on data from:					Predictions on data from:			
C-index	Q2	Q3	Q4	Q5	C-index	Q2	Q3	Q4	Q5
$\xi = 0.50$	0.82	0.82	0.81	0.84	$\xi = 0.50$	0.82	0.81	0.81	0.81
$\xi = 0.75$	0.82	0.82	0.81	0.84	$\xi = 0.75$	0.82	0.82	0.81	0.81
$\xi = 0.90$	0.82	0.82	0.81	0.84	$\xi = 0.90$	0.82	0.82	0.81	0.81
$\xi = 0.99$	0.82	0.82	0.81	0.85	$\xi = 0.99$	0.82	0.82	0.81	0.81
Calibration intercept	Q2	Q3	Q4	Q5	Calibration intercept	Q2	Q3	Q4	Q5
$\xi = 0.50$	-0.13	-0.03	0.02	-0.01	$\xi = 0.50$	-0.01	-0.16	-0.26	-0.36
$\xi = 0.75$	-0.13	-0.03	0.01	-0.03	$\xi = 0.75$	-0.01	-0.17	-0.26	-0.37
$\xi = 0.90$	-0.13	-0.03	0.01	-0.04	$\xi = 0.90$	-0.01	-0.17	-0.27	-0.37
$\xi = 0.99$	-0.13	-0.03	0.00	-0.04	$\xi = 0.99$	-0.01	-0.17	-0.27	-0.37
Calibration slope	Q2	Q3	Q4	Q5	Calibration slope	Q2	Q3	Q4	Q5
$\xi = 0.50$	0.97	0.98	0.92	0.94	$\xi = 0.50$	1.01	1.01	1.00	1.00
$\xi = 0.75$	0.97	0.98	0.94	0.95	$\xi = 0.75$	1.01	1.01	1.00	1.01
$\xi = 0.90$	0.97	0.98	0.94	0.96	$\xi = 0.90$	1.01	1.01	1.00	1.01
$\xi = 0.99$	0.97	0.98	0.94	0.96	$\xi = 0.99$	1.01	1.01	1.00	1.01
Brier score	Q2	Q3	Q4	Q5	Brier score	Q2	Q3	Q4	Q5
$\xi = 0.50$	0.01	0.01	0.01	0.00	$\xi = 0.50$	0.01	0.01	0.01	0.01
$\xi = 0.75$	0.01	0.01	0.01	0.00	$\xi = 0.75$	0.01	0.01	0.01	0.01
$\xi = 0.90$	0.01	0.01	0.01	0.00	$\xi = 0.90$	0.01	0.01	0.01	0.01
$\xi = 0.99$	0.01	0.01	0.01	0.00	$\xi = 0.99$	0.01	0.01	0.01	0.01

Appendix E: Study population characteristics

Table S9: Characteristics of the total study population (n=1,000,000) at the study period start date. Number (percent) are presented or median (IQR) for age and body mass index. To calculate age, all individuals are assumed to have a birthdate of 1 July.

**Characteristics**		**Total cohort**	
Age (years)	median (IQR)	45	(31-61)
Body mass index (kg/m^2)	median (IQR)	25.5	(22.5-29.2)
	missing	184,213	(18.4)
Sex	Male	497,679	(49.8)
	Female	502,321	(50.2)
Type 1 diabetes	No	994,378	(99.4)
	Yes	5,622	(0.6)
Chronic obstructive pulmonary disease (COPD)	No	977,874	(97.8)
	Yes	22,126	(2.2)
Dementia	No	989,957	(99.0)
	Yes	10,043	(1.0)
Region	East Midlands	24,657	(2.5)
	East of England	37,220	(3.7)
	London	256,070	(25.6)
	North East	22,514	(2.3)
	North West	180,088	(18.0)
	South Central	122,748	(12.3)
	South East	118,690	(11.9)
	South West	99,519	(10.0)
	West Midlands	101,810	(10.2)
	Yorkshire & Humber	36,684	(3.7)

References

R. Bender, T. Augustin, and M. Blettner. Generating survival times to simulate cox proportional hazards models. *Statistics in Medicine*, 24:1713–1723, 2005. doi: 10.1002/sim.2059.

T. H. McCormick, A. E. Raftery, D. Madigan, and R. S. Burd. Dynamic logistic regression and dynamic model averaging for binary classification. *Biometrics*, 68:1–19, 2012. doi: 10.1111/j.1541-0420.2011.01645.x.Dynamic.
